# Assessment of Electrical Resistivity and Oxygen Diffusion Coefficient of Cementitious Materials from Microstructure Features

**DOI:** 10.3390/ma14123141

**Published:** 2021-06-08

**Authors:** Renzhan Zhou, Qiang Li, Jiandong Wang, Kewen Zhou, Rui He, Chuanqing Fu

**Affiliations:** 1 School of Civil and Water Engineering, Bengbu University, Bengbu 232008, China; rzzhou@163.com; 2 College of Civil Engineering and Architecture, Zhejiang University of Water Resources and Electric Power, Hangzhou 310018, China; liq@zjweu.edu.cn; 3 College of Civil Engineering and Architecture, Zhejiang University of Technology, Hangzhou 310034, China; wjd@zjut.edu.cn (J.W.); zhoukewen02@163.com (K.Z.); chuanqingfu@126.com (C.F.); 4 Zhejiang University of Technology Engineering Design Group CO. LTD, Hangzhou 310014, China

**Keywords:** electrical resistivity, non-contact measurement, oxygen diffusion coefficient, capillary porosity

## Abstract

A newly proposed modified non-contact electrical resistivity measurement was used to test the resistivity of concrete and cement mortar. The oxygen diffusion coefficients of concrete and mortar were determined by a gas diffusion measurement, and the capillary porosity of concrete and cement mortar was measured by mercury intrusion porosimetry (MIP) measurement. The obtained electrical resistivity and capillary porosity results were verified with other researchers’ data, the measured electrical resistivity results can be estimated by a simple equation from the capillary porosity results. The obtained oxygen diffusion coefficient results were quantitatively correlated with capillary porosity and electrical resistivity measurement results. The proposed equations can be practically used to assess the electrical resistivity and oxygen diffusion coefficient.

## 1. Introduction

The durability of concrete structures is mainly dependent upon its resistance to ingress of aggressive fluids or gases into the micropores in concrete. In general, cementitious materials are considered as porous composites, the interconnected pore structures are of great importance on their durability performance. The pore structure of cementitious materials determine the ingress of chloride [1,2,3], moisture [4,5], gas [6,7] and other ingredients, which may lead to corrosion of internal steel and thus cause service life reduction [8,9,10,11,12,13]. Therefore, the study on the pore structure of cement-based materials after maturity (28 days) is of great significance to the study of structural degradation mechanisms and the prediction of service life.

The electrical resistivity of cementitious materials is an important durability index since it can be directly related with the chloride transportation performance [1,2] and the microstructure [14] of concrete structures. There are many electrical resistivity measurements have been proposed. The most widely used methods include the Wenner method [15,16] and the method specified in ASTM C1202 [17]. The presence of metallic electrodes and the direct current usage bring two major concerns: (1) the polarization effect resulted from the DC current will influence the measurement results; (2) The poor contact between the metallic electrodes and the material will greatly influence the test [18]. A high frequency alternating current was adopted by some researchers to eliminate the polarization problem [19,20,21]. Nevertheless, the metallic electrodes still can cause the contact issue and the electrolysis of water can release gas which would affect the precision of the measurement [22]. To address the polarization and poor contact issues, Li et al. [22,23,24] developed a contactless cementitious materials’ electrical resistivity measurement apparatus. The device has been successfully applied to study the hydration mechanism under different curing conditions [25,26]. This device is proposed in the principle of transformer without the usage of electrodes [27]. An improvement on the non-contact electrical resistivity measurement has been made by previous researchers [14], the modified non-contact electrical resistivity can be used to measure the electrical resistivity of hardened cementitious materials without the presence of electrodes.

For cementitious materials, the gaseous-effective diffusion coefficient is of great importance to describe diffusion-based properties such as carbonation (diffusion of CO_2_ [28]), drying (diffusion of moisture [29]) and corrosion ([30]). It is worth noting that most of laboratory studies focus on gas permeability [31]. However, for concrete structures’ service life prediction model, the gas diffusion coefficient is the most relevant input parameter [31]. The determination of oxygen diffusion coefficient of concrete is challenging due to the high airtightness requirement of the measurement apparatus. An innovative gas diffusion measurement setup was proposed recently [32] which can be used to test the oxygen diffusion coefficient based on Fick’s law. In this work, the capillary porosity of concrete and cement mortar was measured by mercury intrusion porosimetry (MIP) method, the electrical resistivity of saturated cement mortar and concrete was determined by the modified non-contact electrical resistivity measurement and the oxygen diffusion coefficient was measured by the oxygen diffusion measurement. The relationship between the microstructure, electrical resistivity and oxygen diffusion coefficient was proposed. Based on the proposed relationships, the electrical resistivity and oxygen diffusion coefficient can be assessed by the microstructure properties.

## 2. Experimental Program

### 2.1. Materials and Mixture Design

All tests were carried out using ordinary (ASTM C150 [33] Type I) Portland cement as the only binder. The chemical composition and physical properties of the cement are listed in Table 1. The natural sand was used as fine aggregate with an absorption value of 2.15%, and the saturated surface dry (SSD) specific gravity of the fine aggregate was 2.614. The coarse aggregate used was stone with an absorption value of 1.02% and the SSD specific gravity of coarse aggregate was 2.674. All aggregates were oven dried (OD) before mixing, so the moisture value of aggregates was 0.0%. The details of the mixture proportions are given in Table 2, no chemical admixture was used.

### 2.2. Sample Preparation

Three duplicates were cast for each mixture with dimensions of 150 mm × 150 mm × 550 mm. The freshly mixed samples were placed in room condition at 23 ± 1 °C. After 24 h, the samples were de-molded and cured at 23 ± 1 °C, 95% relative humidity (RH) for 28 days.

**Figure 1:**
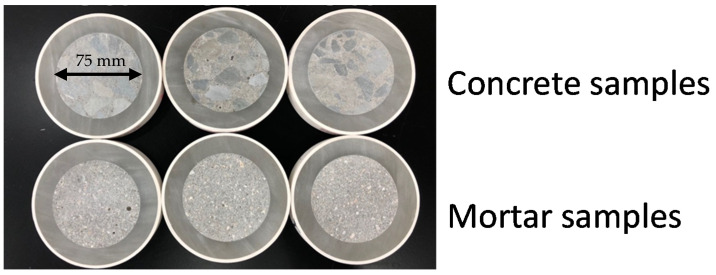
Slice samples.

Cylindrical cores with the dimensions of 75 mm (diameter) and 150 mm (height) were drilled out by a core drilling machine from the casted samples. Each mixture was prepared with 3 cylindrical core samples at 28 days age. Then, cylindrical core samples were put in a PVC pipe with the diameter of 110 mm, the gap between core sample and PVC pipe was filled with fast hardening epoxy. When the epoxy was hardened, a slice sample will be cut from the middle portion of the core sample with the thickness of 20 mm. For each mixture, 6 duplicate slice samples were prepared. The air voids inside the cementitious materials can affect the electrical resistivity measurement results if sample is thinner than 10 mm, so the 20 mm thickness samples were used in this work [34]. The slice samples for modified non-contact electrical resistivity measurements are shown in Figure 1.

All slice samples were pre-oven dried at 60 °C for 48 h to completely remove the free water. For each mixture, 3 oven dried slice samples were used for oxygen diffusivity measurement and the lefted 3 slice samples were used for electrical resistivity measurement. Before electrical resistivity measurement, the slice samples were saturated by 3.5% NaCl solution by a vacuum chamber [35].

The remaining of the cylindrical core samples were cut into pieces and oven dried at 60 °C for 48 h. These samples were used for MIP tests. Each mixture was prepared with 3 samples for MIP measurements.

### 2.3. Modified Non-Contact Electrical Resistivity Measurement

#### 2.3.1. Test Principal

**Figure 2:**
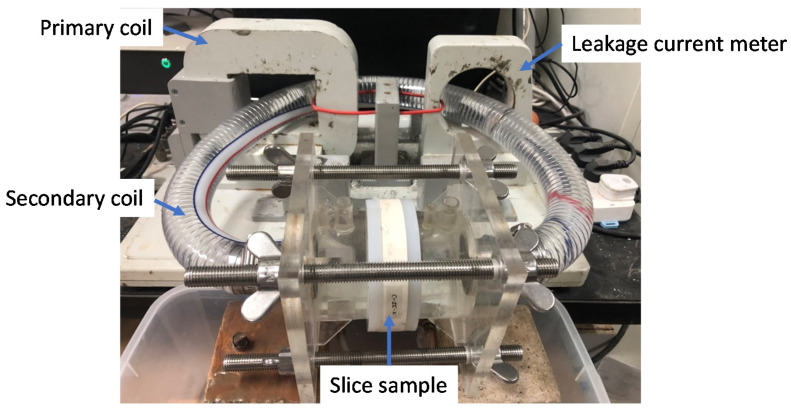
The modified non-contact electrical resistivity measurement setup.

The modified non-contact electrical resistivity measurement setup is presented in Figure 2. The system was composed with a computer, sample platform and mainframe. The computer was used for data collection and the sample platform was consisted with two flanges, solution chamber and solution connecting pipe. During measurement, both sides of the tested sample were clamped by the flanges and fixed with steel bars. The solution chambers were connected by the solution connecting pipe through the transformer core and leakage current meter.

The primary coil of the transformer was composed by a wirewound and the connecting pipe, NaCl solution together with the tested sample were act as the secondary coil of the transformer. During measurement, a 1000 Hz alternating current was applied in to the wirewound coil, then a toroidal current would be generated in secondary coil. The generated toroidal current could be detected and measured by the leakage current meter. The modified non-contact electrical resistivity measurement has been calibrated in [14] by measuring the electrical resistivity of 71.14% KCl solution at 25 °C. The determined electrical resistivity of the solution was 11.626 Ωm, while the quoted value in chemistry handbook [35] was 11.129 Ωm. The relative difference was 4.27%, which indicates the proposed modified non-contact electrical resistivity measurement was highly accurate in determining electrical resistivity. The modified non-contact electrical resistivity measurement has been used to study the pore connectivity [14] and chloride diffusion coefficient [1,2] of cementitious materials.

#### 2.3.2. Test Procedure

**Figure 3:**
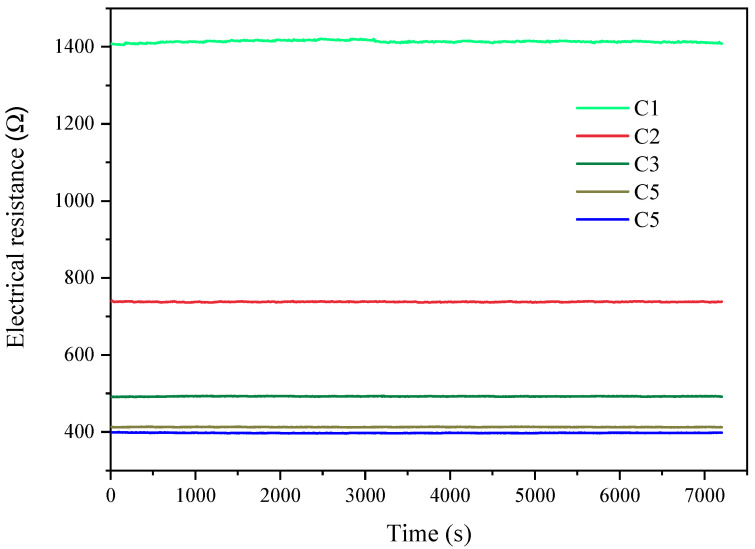
The stability of the measurement (C1 to C5 denote the concrete samples).

Once the measurement was initiated, the computer would automatically record the resistance of the secondary coil at an interval of 10 s and the measurement would be terminated after 2 h. Examples of the recorded resistance results for concrete sample measurements are presented in Figure 3. It is obviously that the recorded electrical resistance results were highly stable during the measurement duration which indicate that slice samples were fully saturated with NaCl solution, and the test was highly stable.

The overall resistance of sample together with 3.5% NaCl solution *R_i_* is the average value of tested, and the resistivity of sample can be calculated based on Ohm’s law in Equation (1):

(1) $$\rho_{i}=\frac{R_{i}-R_{0}}{S_{i}/L_{i}}=\frac{4L_{i}\left(R_{i}-R_{0}\right)}{\pi D_{i}^{2}}$$

where *R_0_* is the resistance of 3.5% NaCl solution, *L_i_* is the thickness of slice sample, *S_i_* is the cross-section area of slice sample, *D_i_* is the diameter of slice sample.

### 2.4. Oxygen Diffusion Measurement

**Figure 4:**
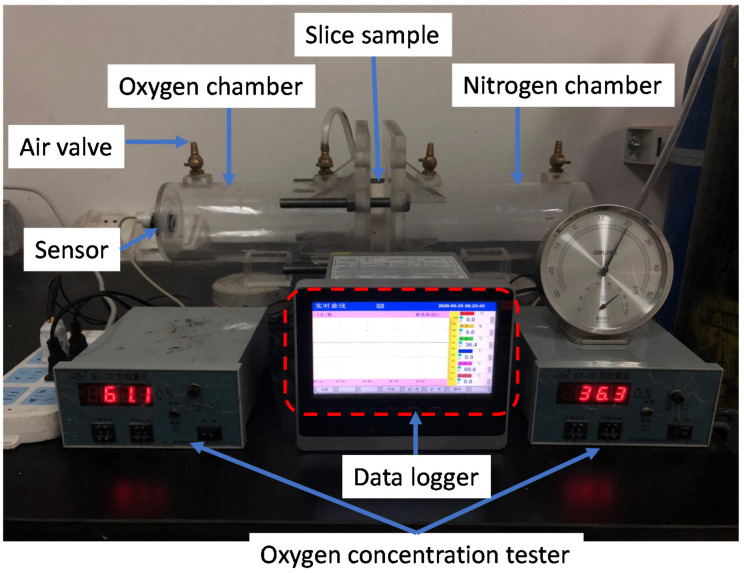
Oxygen diffusion measurement setup.

The oxygen diffusion measurement setup is presented in Figure 4. The tested slice sample was fixed between two air chambers. Before test, both chambers were evacuated into vacuum. Once the measurement initiated, one chamber was filled with pure oxygen and the other chamber was inflated with pure nitrogen simultaneously through the air valve. Once the oxygen concentration in oxygen chamber reaches 95% and nitrogen concentration in nitrogen chamber reaches 95%, the air valves for both chambers were closes. The oxygen concentration sensor will continuously record the oxygen concentration in both chambers.

**Figure 5:**
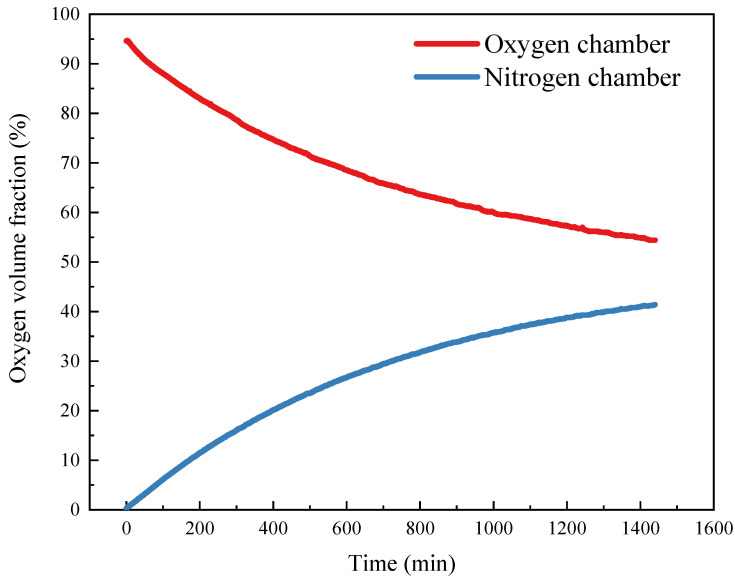
Oxygen volume fraction change in terms of diffusion time in two gas chambers.

The oxygen diffusion coefficient (D_O_) can be calculated by Fick’s law [32] as expressed in Equation (2):

(2) $$D_{o}=\frac{N}{\left(\int_{0}^{t}\frac{\partial C}{\partial L}dt\right)A}$$

where *N* denotes the oxygen flux during the test (mol/m^2^/s), at a given time, $\frac{\partial C}{\partial L}=\frac{C_{0}-C_{1}}{L}$, *C_0_* denotes the oxygen concentration in oxygen chamber and *C*_1_ represents the oxygen concentration in nitrogen chamber (mol/m^3^), the oxygen concentration change in terms of diffusion time is presented in Figure 5, *L* is the slice sample thickness (0.2 m), $\int_{0}^{t}\frac{\partial C}{\partial L}$ is the oxygen concentration gradient in integral of time which can be determined by integrating the fitting of $\frac{\partial C}{\partial L}$ and time. *A* is the cross-section area of the slice sample.

### 2.5. MIP Test

MIP is the most widely adopted measurement for pore structure characterization of porous materials [25,36,37]. This method assumes the pore geometry as cylindrical and the pore size *d* can be directly derived from the applied pressure *P* in accordance with Washburn equation [38],

(3) $$d=-\frac{4\gamma \cos\theta }{P}$$

where *γ* is the mercury surface tension (0.485 N/m), and *θ* is the contact angle between pore wall and mercury which can be taken as 130°. The MIP tests were conducted in Zhejiang University of Technology by an AutoPore IV 9510. The minimum and maximum pressures were 1.4 kPa and 414 MPa, which corresponding to the maximum and minimum pore sizes of 890 μm and 3 nm, respectively.

## 3. Results and Discussion

### 3.1. Electrical Resistivity and MIP Measurement Results

The electrical resistivity and the porosity results from MIP test are concluded in Table 3. The electrical resistivity and capillary porosity results are the average results of three duplicate samples’ results. The electrical resistivity results decrease with the increasing of design w/c which indicate that the higher w/c composite possesses with more pore channel for ion transportation and pore connectivity is higher. The standard deviation of electrical resistivity measurement results for each mixture is neglectable which indicates the modified non-contact electrical resistivity measurement is highly repeatable. The capillary porosity also decreases with the decreasing of w/c, which is reasonable since the lower w/c mix will leave fewer capillary pores after hydration.

The formation factor, *FF*, is the ratio between overall electrical resistivity of cementitious material and the pore solution, expressed as Equation (4):

(4) $$FF=\frac{\rho }{\rho_{0}}$$

where *ρ* is the slice sample’s electrical resistivity as measured by the modified non-contact electrical resistivity measurement in this work, *ρ_0_* represent the electrical resistivity of pore solution, in this work, the capillary pores have been saturated with 3.5% NaCl solution, the electrical resistivity was pre-determined as 0.193 Ωm.

The value of *FF* depends on the pore connectivity of porous materials [39,40]. So, some studies used *FF* to represent the microstructural properties of porous materials, such as ion permeability and gas transportation [1,41,42]. During the first 7 days of concrete’s age, the value of *FF* can decrease by two orders of magnitude since fast hydration would result in reduction in porosity and pore connectivity in early age [43,44].

A relationship between formation factor and the capillary porosity has been proposed by previous researcher [14] on account of Archie’s law [39] as shown in Equation (5),

(5) $$\phi =0.859FF^{-0.380}$$

**Figure 6:**
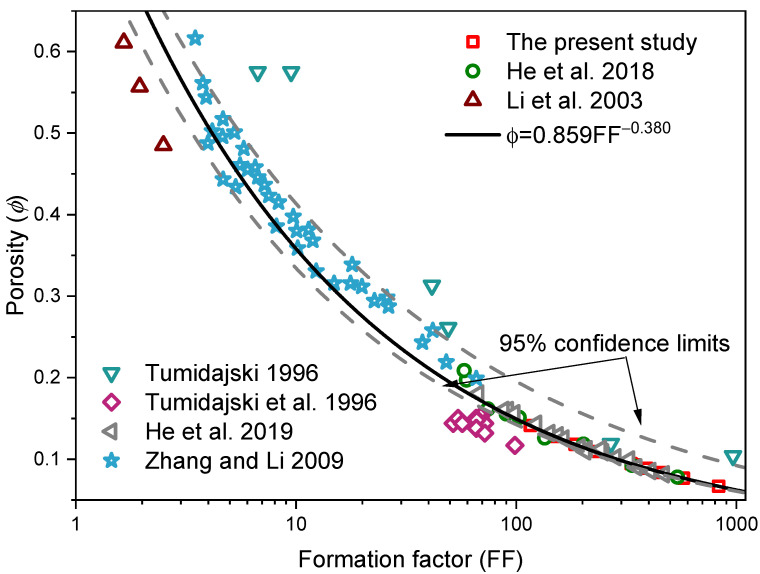
The reasonableness verification of Equation (5).

The reasonableness of this equation can be verified with the experimental results in this work and other researchers’ work [1,14,22,25,45,46] as presented in Figure 6. It can be seen from Figure 6 that Equation (5) is a valid equation in predicting capillary porosity from the measured electric resistivity results.

### 3.2. Oxygen Diffusion Coefficient Determination

**Figure 7:**
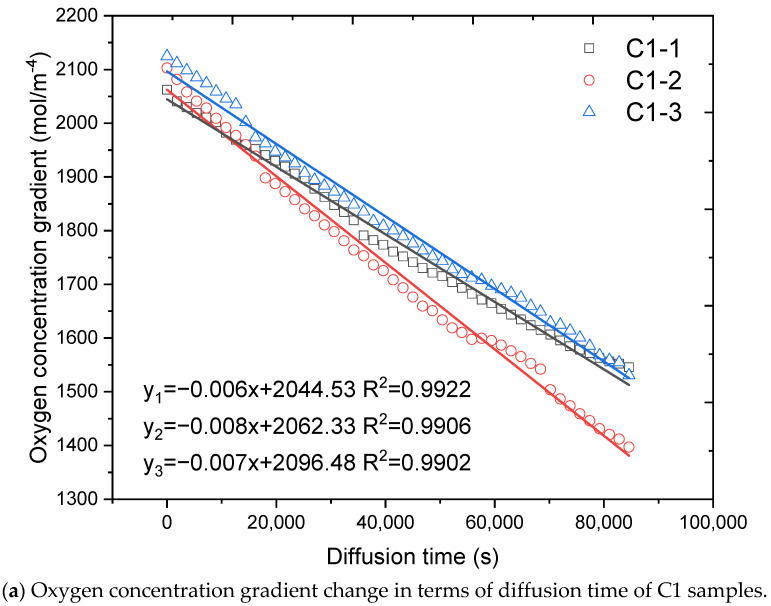

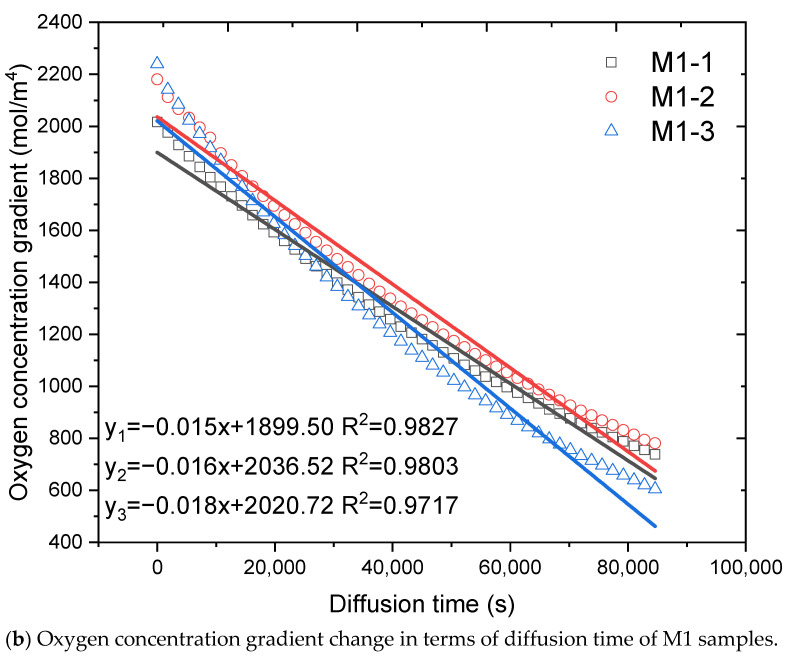
Typical Oxygen concentration gradient change in terms of diffusion time.

The oxygen diffusion coefficient of concrete and cement mortar can be determined from Equation (2) by measuring the oxygen concentration gradient ($\frac{\partial C}{\partial L}=\frac{C_{0}-C_{1}}{L}$) between two gas chambers in function of diffusion time. The typical oxygen gradient between two chambers for concrete and mortar samples are presented in Figure 7. It can be seen that for each sample, the oxygen concentration gradient continuously decreases in terms of diffusion time. For each mixture, three duplicates’ measurement results are highly stable and repeatable.

It reveals that oxygen concentration gradient displayed linear relationship with diffusion time. Hence, linear fitting model (Equation (6)) was selected to for regression analysis. The linear fitting results for C1 samples and M1 samples are presented in Figure 7. It can be observed that the linear regression model shows good fitting results in this work with all fitting parameters R^2^ are higher than 0.9.

(6) $$\frac{\partial C}{\partial L}=At+B$$

where *A* and *B*, are fitting parameters.

The oxygen diffusion coefficients of all samples can be calculated by integral the linear fitting results of oxygen concentration gradient ($\frac{\partial C}{\partial L}$) between two gas chambers in terms of diffusion time by Equation (2). The calculated oxygen diffusion coefficients of all samples are concluded in Table 4.

The standard deviation values in Table 4 of all mixtures are low which indicates the adopted oxygen measurement in this work is highly repeatable. The oxygen diffusion coefficient results lay within the range of 10^−7^ m^2^/s to 10^−8^ m^2^/s as reported by [30]. The oxygen diffusion coefficient results are consistent with expected performance, which is, the higher w/c results in higher oxygen diffusion coefficient.

**Figure 8:**
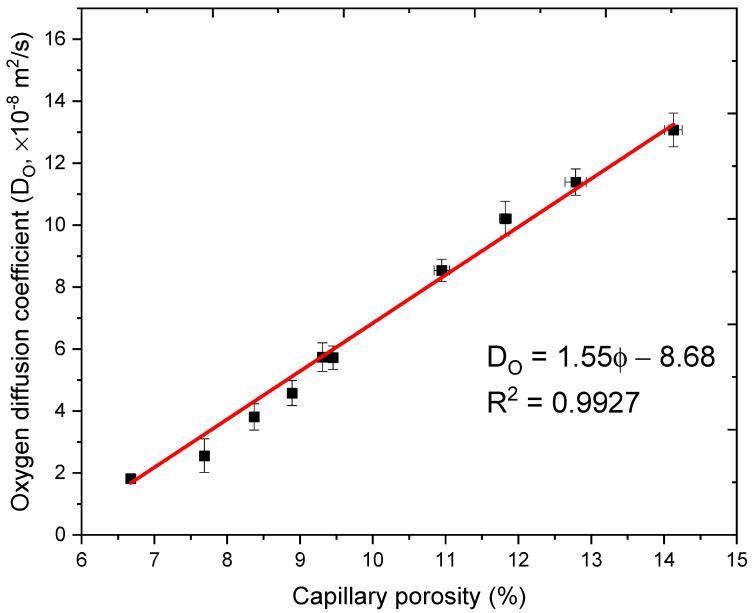
Oxygen diffusion coefficient results in terms of capillary porosity values.

The oxygen diffusion coefficient results in terms of measured capillary porosity values are presented in Figure 8. A linear relation is obtained between the measured oxygen diffusion coefficient results and the capillary porosity results with the fitting parameter R^2^ is as high as 0.9927.

### 3.3. Quantitative Correlation between Porosity, Oxygen Diffusion Coefficient and Electrical Resistivity

The quantitative relations between porosity and oxygen diffusion coefficient, and electrical resistivity can simplify the assessment of gas transportation and ion transportation performance of cementitious materials.

An empirical relationship between diffusion coefficient and electrical resistivity has been proposed by previous researchers [30,47]:

(7) $$D=\frac{n}{\rho }$$

where *D* is the diffusion coefficient (×10^−4^ m^2^/s), *ρ* is the electrical resistivity of cementitious material (×10^−2^ Ωm) and *n* is a constant dependent on the saturation degree and microstructure of cementitious.

**Figure 9:**
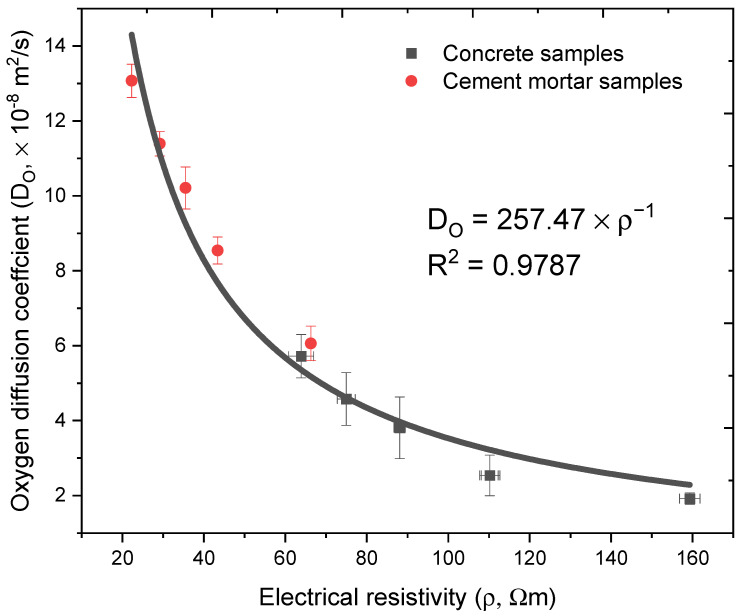
Oxygen diffusion coefficient in terms of electrical resistivity.

The regression result of the experimental results in this work by Equation (7) is presented in Figure 9. The obtained *n* value is 257.47, the regression parameter R^2^ is as high as 0.9787. The obtained *n* value is very close to the results as reported by other researchers in literatures [30,47,48,49].

In this work, as we discussed before, the electrical resistivity of saturated cementitious materials is greatly influenced by the electrical resistivity value of pore solution. Hence, for a more generalized estimation of oxygen diffusion coefficient from electrical resistivity measurement results, Equation (7) can be modified by taking the electrical resistivity into account as:

(8) $$D_{O}=\frac{n\rho_{0}}{FF}$$

The *n* value can also be directly derived by multiply the oxygen diffusion coefficient with the electricity resistivity measurement results as:

(9) $$n=\frac{D_{O}FF}{\rho_{0}}$$

**Figure 10:**
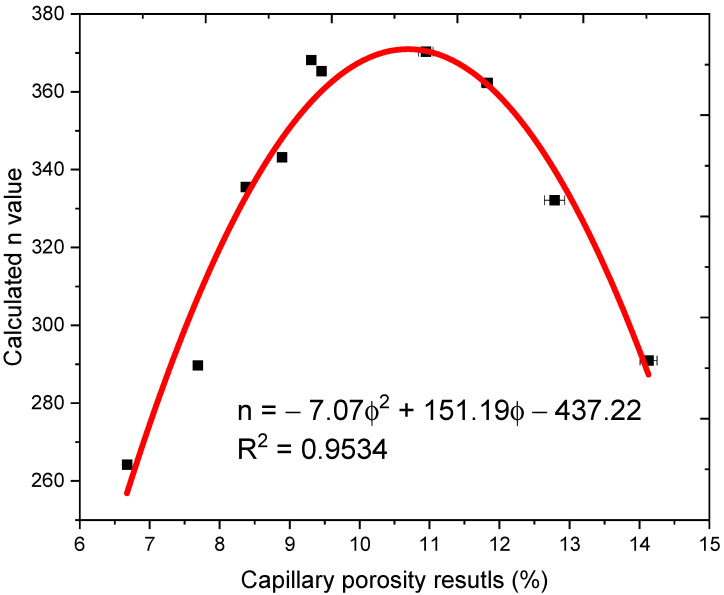
Calculated *n* value in terms of capillary porosity.

The calculated *n* values in terms of measured capillary porosity results are plotted in Figure 10. An empirical equation is obtained to quantitative correlate the *n* value and capillary porosity with the fitting parameter R^2^ is as high as 0.9534.

So, the oxygen diffusion coefficient of cementitious materials can be assessed by the measured porosity and electrical resistivity results as expressed in Equation (10):

(10) $$D_{O}=\frac{-7.07\phi^{2}+151.19\phi -437.22}{FF}\rho_{0}$$

### 3.4. Practical Application

The calculated oxygen diffusion coefficient results by Equation (10) are verified by the measurement results by Equation (11):

(11) $$\Delta D_{O}=D_{O,cal}-D_{O,mea}$$

where Δ*D_O_* is the calculated oxygen diffusion coefficient error (×10^−8^ m^2^/s), *D_O,cal_* and *D_O,mea_* represent the calculated oxygen diffusion coefficient by Equation (10) and the measured oxygen diffusion coefficient, respectively.

**Figure 11:**
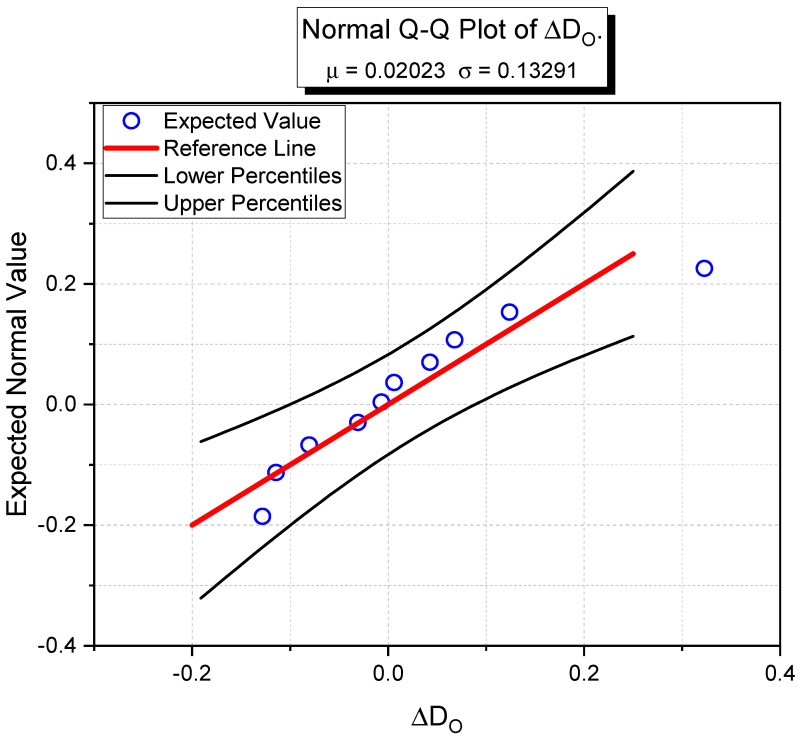
Q-Q plot of calculated oxygen diffusion coefficient error.

The calculated oxygen diffusion error distribution is presented as Q-Q plot with 95% confidence level in Figure 11. The error is significantly drawn in normal distribution population at 5% decision level. Thus, Equation (10) can be a practical model to estimate the oxygen diffusion coefficient of cementitious materials from electrical resistivity and microstructure measurement results.

## 4. Conclusions

In this work, the electrical resistivity values of concrete and cement mortar were measured by a modified non-contact electrical resistivity measurement, the relationship between electrical resistivity, oxygen diffusion coefficient and capillary porosity of cement mortar and concrete was established. The general conclusions can be drawn as:

1. The modified non-contact electrical resistivity measurement can determine the electrical resistivity of cementitious materials, the measurement results is highly stable and repeatable.
2. The universal equation $\phi =0.859FF^{-0.380}$ can be used to estimate the capillary porosity from the measured electrical resistivity results.
3. The oxygen diffusion coefficient of cementitious materials can be assessed from the measured electrical resistivity results and capillary porosity results by the following equation: $D_{O}=\frac{-7.07\phi^{2}+151.19\phi -437.22}{FF}\rho_{0}$.
4. The error of oxygen diffusion coefficient determination results by the proposed assessment equation have 95% confidence to drawn within normality distributed population in 5% decision level, the assessment equation can be practically applied to assess the oxygen diffusion coefficient of cementitious materials.

## Figures and Tables

**Table 1:** Chemical composition (% by mass) and fineness of the cement.

CaO	SiO_2_	Al_2_O_3_	Fe_2_O_3_	MgO	SO_3_	LoI	Fineness (m^2^/kg)
62.91	19.55	5.22	2.74	2.94	3.22	2.25	409

**Table 2:** SSD mixture proportions of cement mortar and concrete (kg/m^3^).

Mixture ID	Design w/c	Cement	Mixing Water	Fine Agg.	Coarse Agg.
C1	0.39	335	130	860	1165
C2	0.42	335	141	849	1150
C3	0.45	335	151	838	1135
C4	0.48	335	161	826	1119
C5	0.51	335	171	815	1104
M1	0.39	686	268	1372	/
M2	0.42	672	282	1345	/
M3	0.45	659	297	1318	/
M4	0.48	646	310	1293	/
M5	0.51	633	323	1267	/

**Table 3:** Electrical resistivity and capillary porosity measurement results.

Mixture ID	Electrical Resistivity (Ωm)	Standard Dev.	Capillary Porosity (%)	Standard Dev.
C1	145.2900	2.5080	6.6733	0.0208
C2	113.1833	2.5007	7.6867	0.0208
C3	88.0767	1.3991	8.3700	0.0200
C4	74.9367	2.2018	8.8900	0.0361
C5	63.8767	3.0608	9.4533	0.0551
M1	64.1451	1.0897	9.3067	0.0513
M2	43.3633	2.0843	10.9467	0.1050
M3	35.4867	1.3466	11.8200	0.0800
M4	29.1600	1.0553	12.7867	0.1457
M5	22.2633	0.4153	14.1300	0.1212

**Table 4:** Oxygen diffusion coefficient results of all mixtures.

Mixture ID	Oxygen Diffusion Coefficient (D_O_ × 10^−8^ m^2^/s)	Standard Dev.
C1 (w/c = 0.39)	1.82	0.1529
C2 (w/c = 0.42)	2.56	0.5427
C3 (w/c = 0.45)	3.81	0.8214
C4 (w/c = 0.48)	4.58	0.7027
C5 (w/c = 0.51)	5.72	0.5784
M1 (w/c = 0.39)	5.74	0.4612
M2 (w/c = 0.42)	8.54	0.3594
M3 (w/c = 0.45)	10.21	0.5594
M4 (w/c = 0.48)	11.39	0.3249
M5 (w/c = 0.51)	13.07	0.4421

## Data Availability

The data presented in this study are available on request from the corresponding author.
